# ENGLISH PROFICIENCY, IMMIGRATION STATUS, AND COGNITIVE ASSESSMENT VARIABILITY IN OLDER ADULTS

**DOI:** 10.1093/geroni/igae098.2099

**Published:** 2024-12-31

**Authors:** Katherine Kitchens

**Affiliations:** The University of Texas Arlington, Arlington, Texas, United States

## Abstract

Recent studies provide evidence that immigration status is a risk factor for developing dementia among older adults and that English proficiency moderates this relationship. This study aimed to analyze the factor structure of the National Health and Aging Trends Study’s (NHATS) cognitive battery to investigate whether this structure varies by English proficiency among the Medicare population and to assess the association between English proficiency, immigration status, and cognition. Utilizing the 2021 NHATS data from a nationally representative sample of Medicare beneficiaries (N = 5,628), a multi-group confirmatory factor analysis (CFA) was used to examine the factor structure of the cognition battery by English proficiency. Three multivariable linear regression models tested the relationship between English proficiency, immigration status, and a standardized cognition score weighted by factor loadings. The multi-group CFA revealed variations in factor loadings and error variances, suggesting differences in how cognition is captured among individuals with varying English proficiency. English proficiency emerged as a significant predictor of better cognitive z-scores, independent of immigration status (b = 0.70, p <.001). Variance in latent cognition construct scores across groups suggests that English proficiency impacts cognitive assessment accuracy, possibly underestimating the cognitive abilities of those with lower English proficiency due to language or cultural barriers. The association between English proficiency, detached from immigration status, counters the perception of immigration status as a cognitive risk factor and calls for cognitive assessments that are culturally and linguistically appropriate and nuanced public health strategies that address diverse immigration experiences and social determinants.

